# 1-[Amino­(4-chloro­phen­yl)meth­yl]-6-bromo­naphthalen-2-ol

**DOI:** 10.1107/S1600536812002905

**Published:** 2012-01-31

**Authors:** A. S. Praveen, H. S. Yathirajan, William T. A. Harrison, Alexandra M. Z. Slawin

**Affiliations:** a Department of Studies in Chemistry, University of Mysore, Manasagangotri, Mysore 570 006, India; b Department of Chemistry, University of Aberdeen, Aberdeen AB24 3UE, Scotland; c School of Chemistry, University of St Andrews, St Andrews KY16 9ST, Scotland

## Abstract

In the title compound, C_17_H_13_BrClNO, the dihedral angle between the naphthol ring system and the chloro­benzene ring is 76.59 (11)°. This twisted conformation is supported by an intra­molecular O—H⋯N hydrogen bond. In the crystal, [100] chains arise, with adjacent mol­ecules linked by an N—H⋯O hydrogen bond, a C—H⋯π inter­action and an aromatic π–π stacking contact [centroid-to-centroid separation = 3.783 (2) Å]. Weak C—H⋯O inter­actions also occur.

## Related literature

For related naphthol–oxazine derivatives and their anti­microbial activity, see: Mayekar *et al.* (2011 ▶).
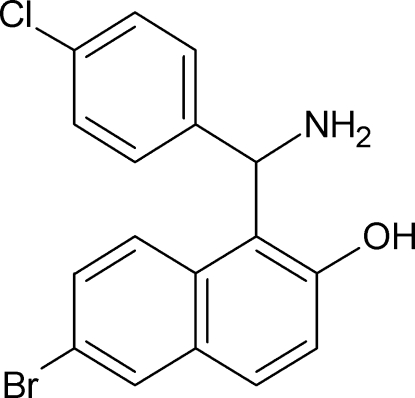


## Experimental

### 

#### Crystal data

C_17_H_13_BrClNO
*M* *_r_* = 361.64
Triclinic, 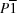

*a* = 4.8026 (15) Å
*b* = 10.785 (3) Å
*c* = 15.086 (4) Å
α = 67.64 (2)°
β = 79.43 (2)°
γ = 85.32 (2)°
*V* = 710.3 (4) Å^3^
*Z* = 2
Mo *K*α radiation
μ = 3.08 mm^−1^
*T* = 73 K
0.12 × 0.10 × 0.10 mm

#### Data collection

Rigaku Mercury CCD diffractometer
Absorption correction: multi-scan (*CrystalClear*; Rigaku, 2009 ▶) *T*_min_ = 0.709, *T*_max_ = 0.748
4392 measured reflections
2426 independent reflections
2222 reflections with *I* > 2σ(*I*)
*R*_int_ = 0.043

#### Refinement

*R*[*F* ^2^ > 2σ(*F* ^2^)] = 0.035
*wR*(*F* ^2^) = 0.082
*S* = 1.05
2426 reflections
199 parameters
H atoms treated by a mixture of independent and constrained refinement
Δρ_max_ = 0.39 e Å^−3^
Δρ_min_ = −0.42 e Å^−3^

### 

Data collection: *CrystalClear* (Rigaku, 2009 ▶); cell refinement: *CrystalClear*; data reduction: *CrystalClear*; program(s) used to solve structure: *SHELXS97* (Sheldrick, 2008 ▶); program(s) used to refine structure: *SHELXL97* (Sheldrick, 2008 ▶); molecular graphics: *ORTEP-3* (Farrugia, 1997 ▶); software used to prepare material for publication: *SHELXL97*.

## Supplementary Material

Crystal structure: contains datablock(s) global, I. DOI: 10.1107/S1600536812002905/bq2335sup1.cif

Structure factors: contains datablock(s) I. DOI: 10.1107/S1600536812002905/bq2335Isup2.hkl

Supplementary material file. DOI: 10.1107/S1600536812002905/bq2335Isup3.cml

Additional supplementary materials:  crystallographic information; 3D view; checkCIF report

## Figures and Tables

**Table 1:** Hydrogen-bond geometry (Å, °) *Cg*1 is the centroid of the C12–C17 benzene ring.

*D*—H⋯*A*	*D*—H	H⋯*A*	*D*⋯*A*	*D*—H⋯*A*
O1—H1O⋯N1	0.90 (3)	1.76 (3)	2.601 (3)	155 (3)
N1—H2N⋯O1^i^	0.84 (3)	2.26 (3)	3.043 (3)	155 (3)
C8—H8⋯O1^ii^	0.95	2.57	3.510 (4)	171
C11—H11⋯*Cg*1^i^	1.00	2.80	3.682 (3)	148

Symmetry codes: (i) 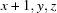; (ii) 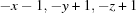.

**Fig. 1.**
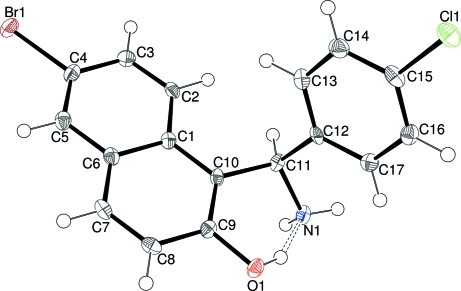
The molecular structure of (I) showing 30% probability displacement ellipsoids for non-H atoms. The O—H···N hydrogen bond is indicated by a double-dashed line.

**Fig. 2.**
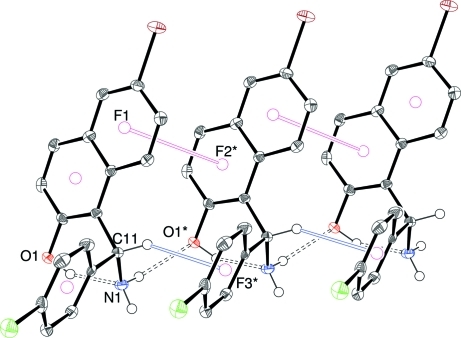
Part of a [100] chain of molecules linked by N—H···O hydrogen bonds (double dashed lines), C—H···π interactions (blue open lines) and aromatic π–π stacking interactions (pink open lines). F1 is the centroid of the C1–C6 ring, F2 is the centroid of the C1/C6–C10 ring and F3 is the centroid of the C12–C17 ring. Atoms with a * suffix are at the symmetry position (x + 1, y, z).

## supplementary crystallographic information

### Comment

As part of our ongoing studies of naphthol–oxazines (Mayekar *et al.*, 2011), we now describe the synthesis and crystal structure of the title compound, (I), (Fig. 1).

The naphthol ring system (C1–C10) in (I) is almost planar (r.m.s. deviation = 0.007 Å) and the Br atom deviates from the mean plane by 0.012 (1) Å. The dihedral angle between the naphthol and chlorobenzene rings is 76.59 (11)°. Atom C11 is a stereogenic centre: in the arbitrarily chosen asymmetric molecule, it has R configuration, but crystal symmetry generates a racemic mixture. The C1—C10—C11—C12 torsion angle is 100.0 (3)° and the twisted conformation of the molecule is supported by an intramolecular O—H···N hydrogen bond (Table 1).

In the crystal, the molecules are linked into [100] chains (Fig. 2), with adjacent molecules linked by an N—H···O hydrogen bond, a C—H···π interaction and a weak π–π stacking contact [centroid–centroid separation = 3.783 (2) Å] between the phenol and bromobenzene rings. A weak C—H···O interaction also occur.

### Experimental

8-Bromo-1,3-bis(4-chlorophenyl)-2,3-dihydro-1H-naphtho[1,2-e][1,3]oxazine (1 mmol) (Mayekar *et al.*, 2011), was suspended in 20% HCl (20 ml) and the mixture was stirred and refluxed for 6 h, whereby the crystalline hydrochloride salt separated out, which was filtered off and washed with ethyl acetate. The solid was suspended in water and the mixture was treated with conc. NH_4_OH (3 ml) and extracted with ethyl acetate. After drying (over anhydrous Na_2_SO_4_) and evaporation of the solvent, the crude product was obtained, which was further purified by recrystallization. Colourless prisms of (I) were grown from the slow evaporation of an ethyl acetate solution (M.p. 413–415 K). Anal. Calcd. for C_17_H_13_BrClNO: C 56.30; H 3.61; N 3.86%; Found: C 56.26; H 3.63; N 3.81%.

### Refinement

The N- and O-bound H atoms were located in a difference map. Their positions were freely refined with the constraint *U*_iso_(H) = 1.2*U*_eq_(N,O) applied. The C-bound H atoms were geometrically placed (C—H = 0.95 Å) and refined as riding with *U*_iso_(H) = 1.2*U*_eq_(C).

### Crystal data

**Table:**

C_17_H_13_BrClNO	*Z* = 2
*M**_r_* = 361.64	*F*(000) = 364
Triclinic, *P*1	*D*_x_ = 1.691 Mg m^−^^3^
Hall symbol: -P 1	Mo *K*α radiation, λ = 0.71073 Å
*a* = 4.8026 (15) Å	Cell parameters from 2706 reflections
*b* = 10.785 (3) Å	θ = 2.0–28.5°
*c* = 15.086 (4) Å	µ = 3.08 mm^−^^1^
α = 67.64 (2)°	*T* = 73 K
β = 79.43 (2)°	Prism, colourless
γ = 85.32 (2)°	0.12 × 0.10 × 0.10 mm
*V* = 710.3 (4) Å^3^	

### Data collection

**Table:**

Rigaku Mercury CCD diffractometer	2426 independent reflections
Radiation source: fine-focus sealed tube	2222 reflections with *I* > 2σ(*I*)
graphite	*R*_int_ = 0.043
ω scans	θ_max_ = 25.0°, θ_min_ = 2.0°
Absorption correction: multi-scan (*CrystalClear*; Rigaku, 2009)	*h* = −5→4
*T*_min_ = 0.709, *T*_max_ = 0.748	*k* = −12→10
4392 measured reflections	*l* = −17→17

### Refinement

**Table:**

Refinement on *F*^2^	Primary atom site location: structure-invariant direct methods
Least-squares matrix: full	Secondary atom site location: difference Fourier map
*R*[*F*^2^ > 2σ(*F*^2^)] = 0.035	Hydrogen site location: inferred from neighbouring sites
*wR*(*F*^2^) = 0.082	H atoms treated by a mixture of independent and constrained refinement
*S* = 1.05	*w* = 1/[σ^2^(*F*_o_^2^) + (0.0351*P*)^2^] where *P* = (*F*_o_^2^ + 2*F*_c_^2^)/3
2426 reflections	(Δ/σ)_max_ < 0.001
199 parameters	Δρ_max_ = 0.39 e Å^−^^3^
0 restraints	Δρ_min_ = −0.42 e Å^−^^3^

### Special details

**Table:**

Geometry. All esds (except the esd in the dihedral angle between two l.s. planes) are estimated using the full covariance matrix. The cell esds are taken into account individually in the estimation of esds in distances, angles and torsion angles; correlations between esds in cell parameters are only used when they are defined by crystal symmetry. An approximate (isotropic) treatment of cell esds is used for estimating esds involving l.s. planes.
Refinement. Refinement of F^2^ against ALL reflections. The weighted R-factor wR and goodness of fit S are based on F^2^, conventional R-factors R are based on F, with F set to zero for negative F^2^. The threshold expression of F^2^ > 2sigma(F^2^) is used only for calculating R-factors(gt) etc. and is not relevant to the choice of reflections for refinement. R-factors based on F^2^ are statistically about twice as large as those based on F, and R- factors based on ALL data will be even larger.

### Fractional atomic coordinates and isotropic or equivalent isotropic displacement parameters (Å^2^)

**Table:**

	*x*	*y*	*z*	*U*_iso_*/*U*_eq_	
C1	0.3027 (6)	0.3901 (3)	0.3133 (2)	0.0134 (6)	
C2	0.5552 (6)	0.3781 (3)	0.2503 (2)	0.0145 (6)	
H2	0.6413	0.4571	0.2020	0.017*	
C3	0.6762 (6)	0.2570 (3)	0.2573 (2)	0.0173 (6)	
H3	0.8447	0.2519	0.2145	0.021*	
C4	0.5505 (6)	0.1395 (3)	0.3281 (2)	0.0166 (6)	
C5	0.3108 (6)	0.1435 (3)	0.3906 (2)	0.0169 (6)	
H5	0.2297	0.0626	0.4380	0.020*	
C6	0.1816 (6)	0.2684 (3)	0.3853 (2)	0.0151 (6)	
C7	−0.0704 (6)	0.2745 (3)	0.4498 (2)	0.0164 (6)	
H7	−0.1529	0.1938	0.4969	0.020*	
C8	−0.1952 (6)	0.3941 (3)	0.4448 (2)	0.0162 (6)	
H8	−0.3644	0.3966	0.4881	0.019*	
C9	−0.0740 (6)	0.5139 (3)	0.3759 (2)	0.0142 (6)	
C10	0.1691 (6)	0.5154 (3)	0.3095 (2)	0.0130 (6)	
C11	0.2826 (6)	0.6491 (3)	0.2339 (2)	0.0145 (6)	
H11	0.4849	0.6360	0.2076	0.017*	
C12	0.1154 (5)	0.7008 (3)	0.1501 (2)	0.0131 (6)	
C13	0.1219 (6)	0.6284 (3)	0.0903 (2)	0.0190 (7)	
H13	0.2285	0.5470	0.1035	0.023*	
C14	−0.0228 (6)	0.6719 (3)	0.0123 (2)	0.0208 (7)	
H14	−0.0159	0.6213	−0.0276	0.025*	
C15	−0.1785 (6)	0.7909 (3)	−0.0065 (2)	0.0177 (6)	
C16	−0.1916 (6)	0.8636 (3)	0.0518 (2)	0.0177 (7)	
H16	−0.3005	0.9443	0.0389	0.021*	
C17	−0.0441 (6)	0.8183 (3)	0.1297 (2)	0.0161 (6)	
H17	−0.0530	0.8689	0.1698	0.019*	
Cl1	−0.35388 (15)	0.84899 (8)	−0.10638 (5)	0.0249 (2)	
Br1	0.72536 (6)	−0.02865 (3)	0.33594 (2)	0.02405 (14)	
O1	−0.2084 (4)	0.6319 (2)	0.37570 (15)	0.0169 (5)	
H1O	−0.070 (6)	0.692 (3)	0.345 (2)	0.020*	
N1	0.2704 (5)	0.7469 (3)	0.2819 (2)	0.0165 (5)	
H1N	0.322 (6)	0.827 (3)	0.240 (2)	0.020*	
H2N	0.387 (6)	0.725 (3)	0.321 (2)	0.020*	

### Atomic displacement parameters (Å^2^)

**Table:**

	*U*^11^	*U*^22^	*U*^33^	*U*^12^	*U*^13^	*U*^23^
C1	0.0138 (14)	0.0130 (14)	0.0145 (16)	−0.0014 (12)	−0.0055 (12)	−0.0047 (12)
C2	0.0184 (15)	0.0143 (15)	0.0103 (15)	−0.0028 (12)	−0.0043 (12)	−0.0025 (12)
C3	0.0168 (15)	0.0195 (15)	0.0168 (17)	0.0000 (13)	−0.0032 (12)	−0.0079 (13)
C4	0.0187 (15)	0.0134 (14)	0.0200 (17)	0.0031 (12)	−0.0079 (13)	−0.0073 (13)
C5	0.0197 (16)	0.0143 (14)	0.0172 (17)	−0.0024 (12)	−0.0062 (13)	−0.0048 (13)
C6	0.0190 (15)	0.0143 (14)	0.0126 (16)	−0.0024 (12)	−0.0059 (12)	−0.0037 (13)
C7	0.0160 (15)	0.0181 (15)	0.0122 (16)	−0.0037 (12)	−0.0034 (12)	−0.0014 (13)
C8	0.0126 (14)	0.0232 (16)	0.0126 (16)	−0.0020 (13)	−0.0019 (12)	−0.0063 (13)
C9	0.0136 (14)	0.0145 (14)	0.0155 (16)	0.0029 (12)	−0.0071 (12)	−0.0054 (13)
C10	0.0143 (14)	0.0104 (13)	0.0137 (15)	0.0002 (11)	−0.0062 (12)	−0.0024 (12)
C11	0.0134 (14)	0.0119 (14)	0.0189 (16)	0.0003 (12)	−0.0034 (12)	−0.0062 (13)
C12	0.0092 (14)	0.0123 (14)	0.0114 (15)	−0.0044 (11)	0.0029 (11)	0.0014 (12)
C13	0.0210 (16)	0.0148 (15)	0.0186 (17)	−0.0001 (13)	−0.0045 (13)	−0.0029 (13)
C14	0.0252 (17)	0.0189 (16)	0.0172 (17)	−0.0056 (14)	0.0001 (13)	−0.0064 (14)
C15	0.0127 (14)	0.0241 (16)	0.0128 (16)	−0.0036 (12)	−0.0036 (12)	−0.0017 (13)
C16	0.0136 (15)	0.0161 (15)	0.0211 (18)	0.0006 (12)	−0.0021 (12)	−0.0051 (13)
C17	0.0157 (15)	0.0162 (15)	0.0168 (16)	−0.0024 (12)	−0.0019 (12)	−0.0065 (13)
Cl1	0.0262 (4)	0.0295 (4)	0.0186 (4)	0.0006 (4)	−0.0098 (3)	−0.0059 (4)
Br1	0.0327 (2)	0.01573 (19)	0.0253 (2)	0.00657 (14)	−0.00654 (15)	−0.00993 (15)
O1	0.0136 (10)	0.0140 (10)	0.0218 (12)	0.0029 (8)	−0.0019 (9)	−0.0063 (9)
N1	0.0194 (14)	0.0112 (12)	0.0198 (15)	0.0006 (11)	−0.0084 (11)	−0.0047 (11)

### Geometric parameters (Å, °)

**Table:**

C1—C2	1.427 (4)	C10—C11	1.525 (4)
C1—C6	1.432 (4)	C11—N1	1.482 (3)
C1—C10	1.434 (4)	C11—C12	1.522 (4)
C2—C3	1.360 (4)	C11—H11	1.0000
C2—H2	0.9500	C12—C17	1.385 (4)
C3—C4	1.403 (4)	C12—C13	1.395 (4)
C3—H3	0.9500	C13—C14	1.384 (4)
C4—C5	1.357 (4)	C13—H13	0.9500
C4—Br1	1.906 (3)	C14—C15	1.390 (4)
C5—C6	1.416 (4)	C14—H14	0.9500
C5—H5	0.9500	C15—C16	1.374 (4)
C6—C7	1.421 (4)	C15—Cl1	1.741 (3)
C7—C8	1.359 (4)	C16—C17	1.391 (4)
C7—H7	0.9500	C16—H16	0.9500
C8—C9	1.403 (4)	C17—H17	0.9500
C8—H8	0.9500	O1—H1O	0.90 (3)
C9—O1	1.378 (3)	N1—H1N	0.88 (3)
C9—C10	1.388 (4)	N1—H2N	0.84 (3)
			
C2—C1—C6	117.0 (2)	C1—C10—C11	122.0 (2)
C2—C1—C10	124.0 (3)	N1—C11—C12	110.7 (2)
C6—C1—C10	118.9 (2)	N1—C11—C10	108.7 (2)
C3—C2—C1	122.0 (3)	C12—C11—C10	111.4 (2)
C3—C2—H2	119.0	N1—C11—H11	108.6
C1—C2—H2	119.0	C12—C11—H11	108.6
C2—C3—C4	119.6 (3)	C10—C11—H11	108.6
C2—C3—H3	120.2	C17—C12—C13	117.9 (3)
C4—C3—H3	120.2	C17—C12—C11	122.8 (2)
C5—C4—C3	121.6 (3)	C13—C12—C11	119.4 (2)
C5—C4—Br1	119.9 (2)	C14—C13—C12	121.7 (3)
C3—C4—Br1	118.5 (2)	C14—C13—H13	119.1
C4—C5—C6	120.0 (3)	C12—C13—H13	119.1
C4—C5—H5	120.0	C13—C14—C15	118.8 (3)
C6—C5—H5	120.0	C13—C14—H14	120.6
C5—C6—C7	120.8 (3)	C15—C14—H14	120.6
C5—C6—C1	119.9 (2)	C16—C15—C14	120.7 (3)
C7—C6—C1	119.3 (2)	C16—C15—Cl1	120.0 (2)
C8—C7—C6	120.9 (3)	C14—C15—Cl1	119.3 (2)
C8—C7—H7	119.5	C15—C16—C17	119.5 (3)
C6—C7—H7	119.5	C15—C16—H16	120.2
C7—C8—C9	120.0 (2)	C17—C16—H16	120.2
C7—C8—H8	120.0	C12—C17—C16	121.3 (3)
C9—C8—H8	120.0	C12—C17—H17	119.3
O1—C9—C10	120.7 (3)	C16—C17—H17	119.3
O1—C9—C8	117.1 (2)	C9—O1—H1O	102.5 (18)
C10—C9—C8	122.2 (2)	C11—N1—H1N	112 (2)
C9—C10—C1	118.6 (3)	C11—N1—H2N	110 (2)
C9—C10—C11	119.4 (2)	H1N—N1—H2N	105 (3)
			
C6—C1—C2—C3	0.4 (4)	C2—C1—C10—C9	−179.1 (3)
C10—C1—C2—C3	179.3 (3)	C6—C1—C10—C9	−0.2 (4)
C1—C2—C3—C4	0.2 (4)	C2—C1—C10—C11	2.1 (4)
C2—C3—C4—C5	−0.4 (4)	C6—C1—C10—C11	−179.0 (2)
C2—C3—C4—Br1	180.0 (2)	C9—C10—C11—N1	43.5 (3)
C3—C4—C5—C6	0.0 (4)	C1—C10—C11—N1	−137.8 (3)
Br1—C4—C5—C6	179.6 (2)	C9—C10—C11—C12	−78.8 (3)
C4—C5—C6—C7	179.6 (3)	C1—C10—C11—C12	100.0 (3)
C4—C5—C6—C1	0.6 (4)	N1—C11—C12—C17	−5.3 (4)
C2—C1—C6—C5	−0.8 (4)	C10—C11—C12—C17	115.8 (3)
C10—C1—C6—C5	−179.8 (2)	N1—C11—C12—C13	174.4 (2)
C2—C1—C6—C7	−179.7 (2)	C10—C11—C12—C13	−64.5 (3)
C10—C1—C6—C7	1.3 (4)	C17—C12—C13—C14	0.7 (4)
C5—C6—C7—C8	−179.9 (3)	C11—C12—C13—C14	−179.1 (3)
C1—C6—C7—C8	−1.0 (4)	C12—C13—C14—C15	0.0 (4)
C6—C7—C8—C9	−0.5 (4)	C13—C14—C15—C16	−0.7 (4)
C7—C8—C9—O1	−179.0 (2)	C13—C14—C15—Cl1	178.3 (2)
C7—C8—C9—C10	1.6 (4)	C14—C15—C16—C17	0.8 (4)
O1—C9—C10—C1	179.3 (2)	Cl1—C15—C16—C17	−178.2 (2)
C8—C9—C10—C1	−1.3 (4)	C13—C12—C17—C16	−0.5 (4)
O1—C9—C10—C11	−1.9 (4)	C11—C12—C17—C16	179.2 (3)
C8—C9—C10—C11	177.5 (2)	C15—C16—C17—C12	−0.2 (4)

### Hydrogen-bond geometry (Å, °)

**Table:**

Cg1 is the centroid of the C12–C17 benzene ring.

**Table:**

*D*—H···*A*	*D*—H	H···*A*	*D*···*A*	*D*—H···*A*
O1—H1O···N1	0.90 (3)	1.76 (3)	2.601 (3)	155 (3)
N1—H2N···O1^i^	0.84 (3)	2.26 (3)	3.043 (3)	155 (3)
C8—H8···O1^ii^	0.95	2.57	3.510 (4)	171
C11—H11···Cg1^i^	1.00	2.80	3.682 (3)	148

Symmetry codes: (i) *x*+1, *y*, *z*; (ii) −*x*−1, −*y*+1, −*z*+1.
